# High Expression of Interleukin-3 Receptor Alpha Chain (CD123) Predicts Favorable Outcome in Pediatric B-Cell Acute Lymphoblastic Leukemia Lacking Prognosis-Defining Genomic Aberrations

**DOI:** 10.3389/fonc.2021.614420

**Published:** 2021-03-16

**Authors:** Zhiheng Li, Xinran Chu, Li Gao, Jing Ling, Peifang Xiao, Jun Lu, Yi Wang, Hailong He, Jianqin Li, Yixin Hu, Jie Li, Jian Pan, Sheng Xiao, Shaoyan Hu

**Affiliations:** ^1^ Institute of Pediatric Research, Children’s Hospital of Soochow University, Suzhou, China; ^2^ Department of Pathology, Brigham and Women’s Hospital, Harvard Medical School, Boston, MA, United States; ^3^ Department of Hematology, Children’s Hospital of Soochow University, Suzhou, China

**Keywords:** B-cell acute lymphoblastic leukemia, pediatric, CD123, prognosis, favorable

## Abstract

**Background:**

Aberrant expression of CD123 (IL-3Rα) was observed in various hematological malignancies including acute lymphoblastic leukemia (ALL), which is the most common malignancy in childhood. Although widely used for minimal residual disease (MRD) monitoring, the prognostic value of CD123 has not been fully characterized in pediatric B-ALL. This retrospective study aims to evaluate the association between the CD123 expression of leukemic blasts and the outcomes of the pediatric B-ALL patients.

**Methods:**

A total of 976 pediatric B-ALL, including 328 treated with CCLG-ALL-2008 protocol and 648 treated with CCCG-ALL-2015 protocol, were recruited in this retrospective study. CD123 expression was evaluated by flow cytometry. Patients with >50, 20–50, or <20% of CD123 expressing blasts were grouped into CD123^high^, CD123^low^, and CD123^neg^, respectively. The correlation between CD123 expression and the patients’ clinical characteristics, overall survival (OS), event-free survival (EFS), and relapse-free survival (RFS) were studied statistically.

**Results:**

Of 976 pediatric B-ALL, 53.4% from the CCLG-ALL-2008 cohort and 49.2% from the CCCG-ALL-2015 cohort were CD123^high^. In the CCLG-ALL-2008 cohort, CD123^high^ was significantly associated with chromosome hyperdiploidy (p < 0.0001), risk stratification (p = 0.004), and high survival rate (p = 0.005). By comparing clinical outcomes, patients with CD123^high^ displayed favorable prognosis, with a significantly better OS (p = 0.005), EFS (p = 0.017), and RFS (p = 0.045), as compared to patients with CD123^low^ and CD123^neg^. The prognostic value of CD123 expression was subsequently confirmed in the CCCG-ALL-2015 cohort. Univariate and multivariate cox regression model analysis showed that high CD123 expression was independently associated with favorable EFS (OR: 0.528; 95% CI: 0.327 to 0.853; p = 0.009) in this cohort. In patients without prognosis-defining genomic abnormalities, high CD123 expression strongly indicated superior survival rates and was identified as an independent prognosis factor for EFS and RFS in both cohorts.

**Conclusions:**

A group of B-ALL lacks prognosis-defining genomic aberrations, which proposes a challenge in risk stratification. Our findings revealed that high CD123 expression of leukemic blasts was associated with favorable clinical outcomes in pediatric B-ALL and CD123 could serve as a promising prognosis predictor, especially in patients without prognosis-defining genetic aberrations.

## Background

Acute lymphoblastic leukemia (ALL) is a common malignancy in childhood, accounting for 25% of cancers in children <15 years of age (1). Advances in diagnosis and risk-directed treatment over the past 50 years have led to remarkable improvements in survival in pediatric ALL, with a long-term survival rate approaching 90% in many developed countries (2). However, the long-term survival rates of pediatric B-ALL in China still fall behind, with nearly 1/3 of the patients failing to achieve long-term disease-free survival. This lag was due to delayed diagnosis, lack of physicians and nurses, inadequate supportive care infrastructure, limited access to effective treatment, high rates of treatment-related mortality, increased relapse, and treatment abandonment in low/medium income countries (3, 4). The rapid development of next generation sequencing prompted the discovery of novel subtypes of B-ALL, such as ph-like, *TCF3*-rearranged, *MEF2D*-rearranged B-ALL (5). However, many hospitals in developing countries currently were unable to identify these novel subgroups owing to lack of advanced sequencing equipment. Thus, this highlights the necessity to identify alternative prognosis predictors to make a better risk stratification and further improve the clinical outcome of pediatric B-ALL patients.

CD123, or the alpha chain of interleukin 3 receptor (IL-3Rα), is a 75 kD glycoprotein encoded by a gene located at the pseudo-autosomal regions of chromosomes Xp22.3 and Yp11.3. By forming a heterodimer receptor with the β common (βc) subunit, CD123 facilitates transmission of IL-3 signaling, which is an important cytokine involved in the regulation of the function and production of hematopoietic and immune cells (6, 7). IL-3Rα binds to IL-3 with high specificity but with low affinity, while the interaction of IL-3Rα and the βc subunit enables high-affinity binding and intracellular signal transduction (6, 8–10).

CD123 is expressed widely in hematological malignancies including acute leukemia, blastic plasmocytoid dendritic cell neoplasm (BPDCN), and hairy cell leukemia (11, 12). Most previous investigations elucidated the role of CD123 in acute myelogenous leukemia (AML). CD123 was highly expressed on AML blasts and correlated with an adverse prognosis (13). More importantly, aberrant CD123 expression was found in AML stem cells (LSCs) (CD34+/CD38-cells) but not in normal bone marrow CD34+/CD38− cells, making it an attractive therapeutic target for AML (14). CD123 was also used as a marker for minimal residual disease (MRD) in AML (15). Recently, anti-CD123 therapeutic strategies, such as CD123 specific antibody and CD123-targeted chimeric antigen receptor (CAR) T cells, have been developed in AML treatment (16, 17).

Similar to AML, CD123 was also identified as a valuable marker for MRD monitoring in ALL (18). Uckun FM et al. has reported early in 1989 that functional CD123 was expressed on the surface of B-ALL (19). Luz Munoz et al. confirmed this finding by flow cytometry showing that all the B-ALL patients were positive in CD123 expression. In contrast, normal lymphoid precursors lacked CD123 expression (11). However, the prognostic relevance of CD123 expression level in pediatric ALL has not been fully characterized. In this retrospective study, we aimed to evaluate the correlation of CD123 level with prognostic significance in pediatric B-ALL patients from our single institution.

## Methods

### Patients Cohorts

A total of 976 B-ALL patients treated with CCLG-ALL-2008 (Chinese Children’s Leukemia Group) or CCCG-ALL-2015 (Chinese Children’s Cancer Group) protocol were included in this retrospective study. B-ALL was diagnosed with a combination of standard cell morphology, immunophenotype, cytogenetics, and molecular testing (MICM) according to the 2008/2017 WHO Classification of Tumors of Hematopoietic and Lymphoid Tissues.

The CCLG-ALL-2008 cohort included 328 pediatric B-ALL treated at Children’s Hospital of Soochow University between December 2008 and October 2015. The last follow-up time was December 2019. The criteria for risk stratification were described previously (20). Patients were treated with risk-based regimens according to the CCLG-ALL-2008 protocol (21).

The CCCG-ALL-2015 cohort included 648 patients with newly diagnosed B-ALL at Children’s Hospital of Soochow University from January 2015 to November 2019. CCCG-ALL-2015 protocol was an MRD-directed and risk-stratified treatment modified from St. Jude Children’s Hospital Total XV study and Shanghai Children’s Medical Center ALL 2005 (4). The latest follow-up time was December 2019.

Both protocols were approved by the Children’s Hospital of Soochow University Institutional Ethics Committee. Informed consent was signed by parents or guardians of each patient.

### Immunophenotyping

Two to three ml of bone marrow with heparin anticoagulation was collected from pediatric patients in the initial diagnosis before chemotherapy. Cells were counted and the cell concentration was adjusted to 10–20 × 10^9^cells/L then followed by a standard stain-lyse-wash procedure. Fifty μl cells (1 × 10^6^) were stained in each analysis tube using CD45-FITC, CD10-PE, CD34-Percp, and CD19-APC monoclonal antibodies in all tubes, with additional antigens conjugated to FITC and phycoerythrin (PE) including CD123, TdT, cyμ, sIgM, CD20, cyCD22, CD22, cyCD79a et al., which were common MRD markers for B-ALL. The CD123 antibody was purchased from BD Biosciences [340545; Clone 9F5 (RUO (GMP); San Diego, CA, USA]. A mouse BALB/c IgG1, κ isotype antibody was used as control [550617; Clone MOPC-31C (RUO); BD Biosciences]. Blasts were gated using side scatter (SSC) *versus* CD45. The expression of CD123 on gated blasts were acquired in Beckman Coulter flow cytometry (Beckman Coulter, USA) and analyzed by BD FACSDiva^TM^ software (BD Biosciences, CA, USA). Blasts with 20% or more expressing CD123 were considered positive.

### Cytogenetic Analysis

Cells from bone marrow aspirate were cultured in RPMI-1640 with 20% FBS for 24 h. Before harvesting, cells were treated with colcemid (0.5 μg/ml) for 20 min. The cells were collected and incubated in hypotonic solution (0.56% KCl), fixed in prechilled fixative (methanol: acetic acid = 3:1), and metaphase slides were manually prepared. After baking at 70°C for 1 h, chromosomes were banded by Giemsa-Trypsin staining.

The presence of certain genomic abnormalities (including the *ETV6/RUNX1*, *BCR/ABL1*, *E2A/PBX1*, and *KMT2A* rearrangement) was analyzed using a B-ALL fluorescence *in situ* hybridization (FISH) panel. Briefly, freshly prepared slides were denatured in 2×SSC at 37°C for 30 min and transferred to ethanol solutions in the order of 70, 80, 90, and 100% ethanol for 2 min each. After drying the slides, 10 μl of the probe was added to the center of the hybridization area on slides and denatured at 80°C for 1 min on a heating block. Hybridization was performed by overnight incubation at 37°C. Slides were washed in 0.4×SSC containing 0.3% NP-40 at 73°C for 3 min and counterstained with DAPI. Fluorescence signaling was observed under the Olympus imaging system.

### Multiplex RT-PCR Analysis

Total RNA from patients’ bone marrow aspirate were isolated by TRIZOL reagent (Invitrogen, Carlsbad, CA, USA). A multiplex reverse transcription polymerase chain reaction was performed to detect common fusion transcripts in B-ALL, including *BCR/ABL1*, *E2A/PBX1*, *ETV6/RUNX1*, *HOX11*, and *KMT2A-* rearrangements. cDNA was synthesized from 1 μg of total RNA as template, 500 ng of six random primers (Promega, USA), 200 U of M-MLV Reverse transcriptase (Promega, USA), and 20 U of RNase inhibitor (Thermo Fisher Scientific, MA, USA) in a total volume of 25 μl. The reaction was incubated at 65°C for 5 min, 37°C for 45 min and terminated at 95°C for 5 min. After the reaction, cDNA product was diluted to 50 µl. Rearrangement detection was performed as eight parallel nested (two-round) multiplex reactions in a Perkin Elmer 9600 thermocycler (Roche Molecular Systems, Branchburg, NJ, USA). Then 5 μl of diluted cDNA was added to each of 50-µl multiplex mixtures which contained 5 μl of 10X buffer, 1 μl of 10 mmol/L dNTP, 5 pmol of each primer, and 1.5 U AmpliTaq-Gold polymerase (Perkin Elmer). The first PCR consisted of an initial activation of the polymerase at 94°C for 5 min, followed by 25 cycles of PCR amplification (annealing at 94°C for 30 s, elongation at 56°C for 30s, and denaturation at 72°C for 60 s). After the first PCR, 1 µl aliquots from each of the eight PCR reactions were transferred to second-round multiplex mixtures. The program setting of PCR cycle was same as the first round PCR. Quality controls included a normal RNA specimen and β-actin amplification. The oligonucleotide primers used in multiplex RT-PCR were provided in the **Supplementary Material** (**Table S1**).

### Treatment Response Assessment

In the CCLG-ALL-2008 study, treatment response assessment included prednisone response, bone marrow remission status on day 15 and day 33 of the induction therapy, and MRD levels on day 33 (after induction) and week 12 (after consolidation). For bone marrow remission status evaluation, M1, M2, and M3 were defined as <5, 5–25, or >25% of blasts in bone marrow, respectively. In the CCCG-ALL-2015 cohort, treatment response was assessed at day 19 and day 46 by MRD measurements.

### Clinical Outcome Assessment

The OS was calculated from the date of diagnosis to the deceased date or the last follow-up date; EFS was estimated from the date of diagnosis until the date of any of the following events occurring: relapse, death, failure to remission, HSCT, or treatment abandonment; RFS was estimated from the date of diagnosis to the date of the first relapse.

### Statistical Analysis

Data were presented as mean value ± standard deviation, median, count, or percentage. Categorical variables were compared by the Chi-square test. Kaplan–Meier method was used to determine the impact of factors on the OS, EFS, and RFS. The log-rank test was used to compare the survival rates among groups. Multivariate analysis was performed using Cox proportional hazards regression model. A *p* value <0.05 was considered statistically significant. All statistical analyses were performed using SPSS Statistic 22.0 software (Armonk, NY, USA: IBM Corp). Graphs were drawn by Graphpad Prism 8.3.0 software (San Diego, CA, USA, www.graphpad.com).

## Results

### Correlation of CD123 Expression With Clinicopathologic Data

The median percentage of CD123-expressing cells was 52.94% (range: 0.1–99.9%) and 48.69% (range: 0.00–99.8%) in the CCLG-ALL-2008 and the CCCG-ALL-2015 cohorts, respectively (**Figure S1**). In order to explore the clinical relevance of CD123 expression in pediatric B-ALL patients, we set the percentage of CD123-expressing blasts at 50% as a cut off value for further analysis. Patients were classified into CD123^high^ (>50% of blasts with CD123 expression), CD123^low^ (20–50% with CD123 expression), and CD123^neg^ (<20% with CD123 expression).

In the CCLG-ALL-2008 cohort, 328 B-ALL patients with a mean age of 5.08 years (range: 6 months–14.83 years) at diagnosis were enrolled, with 190 male (57.9%) and 138 female (42.1%). The median follow-up time was 76 months (range: 50–136 months). The mean value of WBC count at initial diagnosis was 39.31 (range: 0.39–705.72) × 10^9^ cell/L. CD123 was expressed in 75% (246/328) of patients, with 71 cases (28.9%) being CD123^low^ and 175 cases (71.1%) being CD123^high^. Statistic evaluation showed a significant association between high CD123 expression and chromosome hyperdiploidy (p < 0.0001), lower WBC count (p = 0.015), and standard-risk group among three groups of standard, intermediate, and high risks (p = 0.004). No difference was found between CD123 expression and presenting age (p = 0.539), gender (p = 0.248), sensitivity to steroid treatment (p = 0.634), BM remission status, and MRD levels on Day 15 and Day 33 post-induction (**Table 1**).

**Table 1:** Correlation of CD123 expression with clinicopathological features of pediatric B-ALL patients treated with CCLG-ALL-2008 protocol.

Feature	Category	Total	CD123						p value
			Negative		Low percentage		High percentage		
All		328	82	25.0%	71	21.6%	175	53.4%	
Gender									0.248
	M	190	50	26.3%	35	18.4%	105	55.3%	
	F	138	32	23.2%	36	26.1%	70	50.7%	
Age (years)									0.539
	<1	4	2	25.0%	1	25.0%	2	50.0%	
	1–10	287	68	23.7%	62	21.6%	157	54.7%	
	>10	37	12	32.4%	8	21.6%	17	45.9%	
Chromosome hyperdiploidy*									**<0.0001**
	N	174	54	31.0%	48	27.6%	72	41.4%	
	Y	60	4	6.7%	4	6.7%	52	86.7%	
Molecular abnormality									**<0.0001**
	*ETV6/RUNX1* fusion	61	21	34.4%	19	31.1%	21	34.4%	
	*BCR/ABL1* fusion	14	4	28.6%	6	42.9%	4	28.6%	
	*E2A/PBX1* fusion	22	19	86.4%	3	13.6%	0	0.0%	
	*KMT2A-* rearrangement	9	3	33.3%	3	33.3%	3	33.3%	
	*HOX11*	19	1	5.3%	2	10.5%	16	84.2%	
	Mixed**	1	0	0.0%	0	0.0%	1	100%	
WBC (×10^9^/L)									**0.015**
	WBC<20	199	38	19.1%	43	21.6%	118	59.3%	
	20<WBC<50	60	16	26.7%	15	25.0%	29	48.3%	
	50<WBC<100	37	15	40.5%	6	16.2%	16	43.2%	
	WBC>100	30	13	43.3%	7	23.3%	10	33.3%	
Risk group									**0.004**
	SR	86	13	15.1%	17	19.8%	56	65.1%	
	IR	128	35	27.3%	28	21.9%	65	50.8%	
	HR	106	31	29.2%	24	22.6%	51	48.1%	
Day 15 BM remission^†^									0.068
	M1	164	49	29.9%	36	22.0%	79	48.2%	
	M2	96	16	16.7%	17	17.7%	63	65.6%	
	M3	61	15	24.6%	15	24.6%	31	50.8%	
Day 33 BM remission^‡^									0.599
	N	296	72	24.3%	67	22.6%	157	53%	
	Y	15	5	33.3%	2	13.3%	8	53.3%	
Day 33 MRD level^#^									0.147
	<0.01%	172	43	25.0%	34	19.8%	95	55.2%	
	0.01–1%	111	18	16.2%	25	22.5%	68	61.3%	
	>1%	14	6	42.9%	3	21.4%	5	35.7%	
Week 12 MRD level^##^									0.576
	<0.1%	246	54	22.0%	49	19.9%	143	58.1%	
	>0.1%	31	8	25.8%	8	25.8%	15	48.4%	
Steroid response^§^									0.634
	Sensitive	284	69	24.3%	61	21.5%	154	54.2%	
	Resistant	41	12	29.3%	10	24.4%	19	46.3%	
Relapse									0.051
	N	272	62	22.8%	57	21.0%	153	56.3%	
	Y	56	20	35.7%	14	25.0%	22	39.3%	
Death									**0.005**
	N	279	66	23.7%	54	19.4%	159	57.0%	
	Y	49	16	32.7%	17	34.7%	16	32.7%	

*No metaphase cell available for karyotyping in 94 patients.

**One case with KMT2A/AF10 rearrangement and HOX11.

^†^Patients lacking D15 BM status data were excluded.

^‡^Patients lacking D33 BM status data were excluded.

^#^No specific marker suitable for MRD monitoring in seven patients.

^##^No specific marker suitable for MRD monitoring in seven patients and no MRD data available in 10 patients.

^§^Undetermined drug response in three patients.

WBC, white blood cell; SR, standard risk; IR, intermediate risk; HR, high risk; MRD, minimal residual disease. Bold numbers represent that p value reaches statistical significance.

In the CCCG-ALL-2015 cohort, 648 patients with a median age of 5.05 years (range: 7 months–16.58 years) were enrolled. The median follow-up time was 25.86 months (range: 1–59 months). The male-to-female ratio was 1.3:1 (366:282). Of the 648 cases, 439 (67.7%) patients had a positive CD123 expression. The significant correlation of CD123^high^ with chromosome hyperdiploidy (p < 0.0001), and standard-risk group (p = 0.004) were confirmed in this cohort (**Tables S1** and **S2**). There was no significant difference in CD123 percentage with age (p = 0.519), gender (p = 0.567), and treatment response, which includes sensitivity to steroid treatment (p = 0.912), MRD levels at day 19 (p = 0.363), and day 46 (p = 0.902). However, differing from in the CCLG-ALL-2008 cohort, no significant difference was found between the WBC count and CD123 expression in patients treated with the CCCG-ALL-2015 protocol (p = 0.344).

### Correlation of CD123 Percentage With Clinical Outcomes

In the CCLG-ALL-2008 cohort, mortality was significantly lower in patients with high CD123 expression (9.1%, 16/175) as compared to those with lower CD123 expression (23.9%, 17/71) or negative CD123 expression (19.5%, 16/82; p = 0.005). The incidence of relapse was also lower in patients with high CD123 expression (12.6%, 22/175) as compared to those with lower CD123 expression (19.7%, 14/71) or negative CD123 expression (24.39%, 20/82), although the difference did not reach statistically significant (p = 0.051) (**Table 1**). Similarly, patients in the CD123^high^ group showed a significantly lower risk of mortality (p = 0.039) and relapse (p = 0.044) in the CCCG-ALL-2015 cohort (**Tables S1** and **S2**).

**Figure 1:**
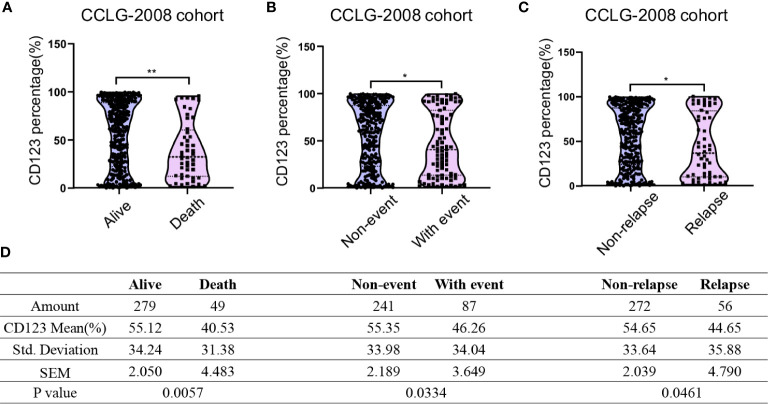
Pediatric B-ALL with favorable clinical outcomes displayed high CD123 expression in the CCLG-ALL-2008 cohort. Comparison of CD123 expression in leukemic blasts between survivors and non-survivors **(A)**, event-free and with-event patients (events including drug resistance, treatment abandonment, relapse, and HSCT) during treatment **(B)**, and relapsed or non-relapsed B-ALL **(C)** in the CCLG-ALL-2008 cohort; **(D)** Detailed amounts of patients and mean CD123 percentage in each group from A to C *p < 0.05; **p < 0.01.

We next compared the CD123 expression between survivors and non-survivors with B-ALL. In the CCLG-ALL-2008 cohort, a significantly higher mean CD123 expression (55.12 ± 2.05%) was found in survivors as compared to the non-survivors (40.53 ± 4.483%; p = 0.0057) (**Figures 1A, D**). The therapeutic outcome was complicated by the occurrence of events such as drug resistance, treatment abandonment, and disease progress. Compared with patients with events during treatment (46.26 ± 3.649%), higher CD123 expression was shown in event-free patients (55.35 ± 2.189%; p = 0.0334) (**Figures 1B, D**). Furthermore, a significantly higher CD123 expression was found in relapse-free patients as compared to that of the relapsed ones (p = 0.0461) (**Figures 1C, D**). A similar association between CD123 expression and therapeutic outcomes was confirmed in patients treated with CCCG-ALL-2015 protocol (**Figure S2**). In this cohort, a higher CD123 expression was seen in the event-free group (50.56 ± 1.524%) as compared to that of the patient with events during treatment (37.89 ± 3.561%; p = 0.0012). Also, non-relapse patients had remarkably higher mean CD123 expression (49.55 ± 1.451%) than that of the relapsed patients (33.69 ± 5.622%; p = 0.0082).

**Figure 2:**
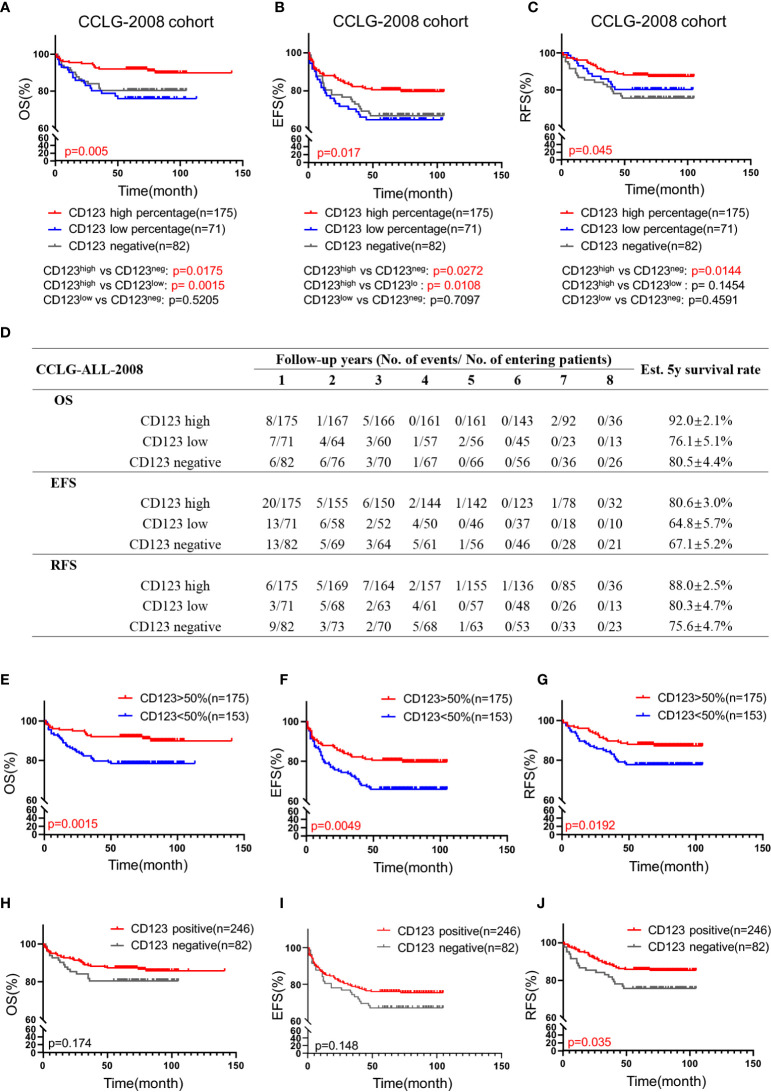
High CD123 expression correlates with favorable clinical outcomes of pediatric B-ALL treated with CCLG-ALL-2008 protocol. Comparison of OS **(A)**, EFS **(B)**, and RFS **(C)** among CD123^high^, CD123^low^, and CD123^neg^ groups in the CCLG-ALL-2008 cohort; **(D)** The detailed number of event occurrences and cases under observation in the CD123^high^, CD123^low^, and CD123^neg^ groups in every follow-up year. Estimated 5-year survival rates in each group were calculated; Comparison of OS **(E)**, EFS **(F)**, and RFS **(G)** between patients with over or less than 50% blasts expressing CD123; Comparison of OS **(H)**, EFS **(I)**, and RFS **(J)** between patients positively or negatively expressing CD123.

We subsequently explored the association of CD123 expression with OS, EFS, and RFS in pediatric B-ALL. In the CCLG-ALL-2008 cohort, the CD123^high^ group showed a considerably better survival rate than the CD123^neg^ and CD123^low^ groups (**Figures 2A–D**). The estimated mean overall survival time was 124.68 ± 2.7 months in the CD123^high^ group, markedly longer than 86.62 ± 4.6 months in the CD123^low^ group and 72.98 ± 4.37 months in CD123^neg^ group (p = 0.005). A significantly longer mean event-free survival time was seen in the CD123^high^ group (83.1 ± 2.6 months) as compared to that in the CD123^low^ group (67.44 ± 4.9 months) and CD123^neg^ group (72.98 ± 4.37 months; p = 0.017). In addition, patients in the CD123^high^ group showed longer RFS time (90.34 ± 3.9 months) than the other two groups (CD123^low^ group: 83.96 ± 3.7 months; CD123^neg^ group: 80.49 ± 3.9 months; p = 0.045) (**Figures 2A–D**).

Further evaluation between CD123^high^ group (CD123 >50%) and patients with CD123 <50% (combined the CD123^low^ and the CD123^neg^ groups) showed that superior OS, EFS, and RFS were achieved in patients with >50% CD123 expression (OS: p = 0.0015; EFS: p = 0.0049; RFS: p = 0.0192) (**Figures 2E–G**). When the cut-off value was set at 20%, patients with positive CD123 expression (CD123 >20%) had higher RFS than those with negative CD123 expression (CD123 <20%; p = 0.0035). However, the difference in OS and EFS did not reach statistical significance between these two groups (**Figures 2H–J**).

A similar correlation between CD123 percentage and OS, EFS, and RFS was confirmed in the CCCG-ALL-2015 cohort (**Figure S3**). A better EFS was achieved in CD123^high^ patients than that in CD123^low^ and CD123^neg^ group (p = 0.013). The OS and RFS showed the same trend, but not reached significance (**Figures S3A–D**). Comparison between CD123^high^ and CD123^neg^ group showed that patients with high CD123 expression had significant advantage in OS (p = 0.0319), EFS (p = 0.0044), and RFS (p = 0.0224) (**Figures S3A–C**). Similar survival trends were observed when patients were grouped based on CD123 expression >50 or <50% (**Figures S3E–G**). Moreover, it is noted that positive CD123 expression (>20%) strongly indicated better outcomes in patients treated by the CCCG-ALL-2015 protocol (OS: p = 0.0279; EFS: p = 0.016; RFS: p = 0.032) (**Figures S3H–J**). These discrepancies between the two cohorts might result from the differences in the treatment methods between the two protocols. In addition, compared with the CCLG-ALL-2008 cohort, the follow-up time of the CCCG-ALL-2015 cohort was relatively short (50–136 months *vs* 1–59 months), with a proportion of patients still in the course of treatment.

### CD123 as an Independent Predictor of EFS in Pediatric B-ALL

Univariate Cox analysis was performed and results showed that CD123 high was significantly associated with OS, EFS, and RFS in both cohorts (**Tables 2**–**4**). Factors with p value <0.1 were incorporated into the multivariate Cox proportional hazards regression model. As shown in **Tables 2** **–** **4**, CD123^high^ was not an independent prognostic predictor for longer OS (p = 0.369), EFS (p = 0.424), or RFS (p = 0.058) in CCLG-ALL-2008 cohort. However, high CD123 expression was independently correlated with longer EFS (OR: 0.528; 95% CI: 0.327 to 0.853; p = 0.009), but not independently associated with OS (p = 0.085) and RFS (p = 0.054) in the CCCG-ALL-2015 cohort.

**Table 2:** Cox’s proportional hazards regression model analysis of factors affecting overall survival (OS).

CCLG-ALL-2008												
		Univariate Cox’s regression				Multivariate Cox’s regression						
Variate	Category	OR	95% CI		p value	OR		95% CI				p value
			Lower	Upper				Lower		Upper		
CD123	Negative				0.020							**0.007**
	High	0.573	0.347	0.947	0.030	0.703		0.326		1.516		0.369
Age	<1				0.008							0.122
	1–10	0.182	0.044	0.757	0.019	0.398		0.085		1.866		0.242
WBC (×10^9^/L)	WBC<20				0.004							0.084
	50<WBC<100	3.073	1.438	6.568	0.004	2.659		1.088		6.503		**0.032**
	WBC>100	3.329	1.515	7.314	0.003	2.710		1.109		6.620		**0.029**
Steroid resistance		2.067	1.028	4.157	0.042	1.152		0.487		2.727		0.747
Risk group	LR				0.008							0.749
	HR	3.677	1.502	8.999	0.004	1.523		0.512		4.533		0.450
**CCCG-ALL-2015**												
		**Univariate Cox’s regression**				**Multivariate Cox’s regression**						
**Variate**	**Category**	**OR**	**95% CI**		**p value**	**OR**		**95% CI**				**p value**
			**Lower**	**Upper**				**Lower**		**Upper**		
CD123	High	0.358	0.134	0.956	0.041	0.379		0.126		1.142		0.085
Age	<1				0.070							0.051
	1–10	0.094	0.012	0.734	0.024	0.065		0.06		0.663		**0.021**
Risk group	LR				0.006							0.270
	IR	3.708	1.333	10.314	0.012	3.034		0.791		11.635		0.105
	HR	11.706	2.265	60.506	0.003	0.002		0.000		2.515E+150		0.971
WBC (×10^9^/L)	WBC<20				0.046							0.173
	WBC>100	4.732	1.522	14.710	0.007	2.672		0.737		9.693		0.135
Day 19 MRD level	Positive	2.524	1.015	6.278	0.046	1.415		0.453		4.426		0.551
Day 46 MRD level	Positive	7.137	1.642	31.024	0.009	5834.116		0.000		9.122E+156		0.962

Bold numbers represent that p value reaches statistical significance.

**Table 3:** Cox’s proportional hazards regression model analysis of factors affecting event-free survival (EFS).

CCLG-ALL-2008									
		Univariate Cox’s regression				Multivariate Cox’s regression			
Variate	Category	OR	95% CI		p value	OR	95% CI		p value
			Lower	Upper			Lower	Upper	
CD123	Negative				0.02				0.137
	High	0.573	0.347	0.947	0.030	1.369	0.638	2.936	0.420
WBC (×10^9^/L)	WBC<20				0.016				0.277
	50<WBC<100	1.893	1.039	3.450	0.037	1.315	0.544	3.177	0.543
	WBC>100	1.893	1.265	4.201	0.006	1.542	0.638	3.728	0.336
Steroid resistance		2.529	1.515	4.219	0.000	1.554	0.675	3.580	0.301
Risk group	LR				0.000				0.319
	HR	3.494	1.883	6.481	0.000	1.494	0.545	4.097	0.436
Day 15 BM remission	M1				0.012				0.459
	M3	1.980	1.184	3.310	0.009	0.998	0.434	2.294	0.997
Day 33 MRD level	<0.01%				0.000				0.118
	0.01-1%	2.130	1.285	3.532	0.003	1.980	1.008	3.888	**0.047**
	>1%	5.302	2.404	11.693	0.000	1.267	0.308	5.217	0.743
Week 12 MRD level	>0.1%	3.016	1.621	5.612	0.000	1.985	0.940	4.692	0.118
**CCCG-ALL-2015**									
		**Univariate Cox’s regression**				**Multivariate Cox’s regression**			
**Variate**	**Category**	**OR**	**95% CI**		**p value**	**OR**	**95% CI**		**p value**
			**Lower**	**Upper**			**Lower**	**Upper**	
CD123	Negative				0.016				**0.025**
	High	0.525	0.335	0.822	0.005	0.528	0.327	0.853	**0.009**
WBC (×10^9^/L)	WBC<20				0.002				0.426
	20<WBC<50	1.773	1.045	3.007	0.034	1.365	0.769	2.421	0.288
	WBC>100	3.158	1.693	5.892	0.000	1.368	0.681	2.747	0.378
Steroid resistance		64.206	21.946	187.842	0.000	6.959	1.770	27.365	**0.005**
Risk group	LR				0.000				**0.000**
	IR	2.130	1.379	3.288	0.001	1.902	1.094	3.307	**0.023**
	HR	21.170	10.809	41.462	0.000	18.213	6.575	50.450	**0.000**
Day 19 MRD level	Positive	2.293	1.515	3.472	0.000	1.129	0.646	1.973	0.670
Day 46 MRD level	Positive	21.397	11.388	40.200	0.000	n.s.*			

*Degree of freedom reduced because of constant or linearly dependent covariates. Bold numbers represent that p value reaches statistical significance.

**Table 4:** Cox’s proportional hazards regression model analysis of factors affecting relapse-free survival (RFS).

CCLG-ALL-2008									
		Univariate Cox’s regression				Multivariate Cox’s regression			
Variate	Category	OR	95% CI		p value	OR	95% CI		p value
			Lower	Upper			Lower	Upper	
CD123	Negative				0.051				0.159
	High	0.476	0.260	0.872	0.016	0.513	0.238	1.106	0.088
Risk group	HR	2.165	0.997	4.702	0.051	1.497	0.555	4.041	0.426
Day 33 MRD level	<0.01%				0.002				**0.037**
	0.01–1%	3.110	1.654	5.847	0.000	2.419	1.159	5.047	**0.019**
Week 12 MRD level	>0.1%	2.628	1.254	5.506	0.010	2.075	0.827	5.203	0.120
**CCCG-ALL-2015**									
		**Univariate Cox’s regression**				**Multivariate Cox’s regression**			
**Variate**	**Category**	**OR**	**95% CI**		**p value**	**OR**	**95% CI**		**p value**
			**Lower**	**Upper**			**Lower**	**Upper**	
CD123	Negative				0.080				0.156
	High	0.436	0.211	0.900	0.025	0.470	0.218	1.014	0.054
WBC	WBC<20				0.000				**0.000**
	20<WBC<50	2.706	1.183	6.194	0.018	2.588	1.073	6.242	**0.034**
	WBC>100	7.878	3.527	17.593	0.000	5.883	2.472	14.002	**0.000**
Risk group	LR				0.000				0.066
	IR	4.626	2.181	9.811	0.000	3.067	1.197	7.860	**0.020**
Day 19 MRD level	Positive	2.459	1.274	4.746	0.007	1.726	0.778	3.828	0.179

Bold numbers represent that p value reaches statistical significance.

### Prognosis Value of CD123 for Patients Without Genomic Abnormalities

**Figure 3:**
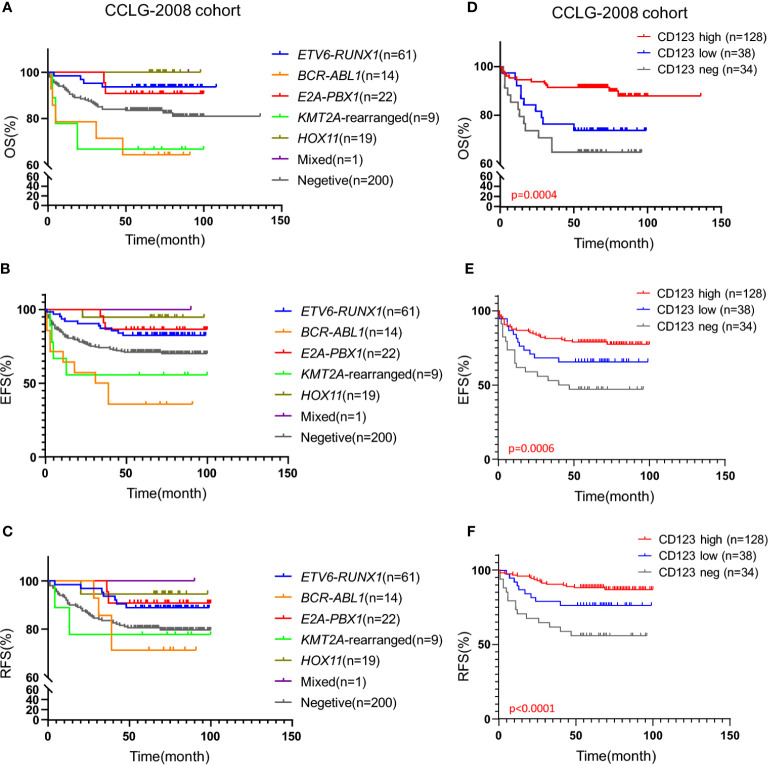
High CD123 expression conferred favorable clinical outcomes in pediatric B-ALL lacking prognosis-defining genomic abnormalities in the CCLG-ALL-2008 cohort. Kaplan-Meier analysis of OS **(A)**, EFS **(B)**, and RFS **(C)** across patients with different genomic abnormalities in the CCLG-ALL-2008 cohort; Comparison of OS **(D)**, EFS **(E)**, and RFS **(F)** according to the CD123 expression among patients without prognosis-defining genetic aberrations in the CCLG-ALL-2008 cohort.

Genomic abnormalities are strong indicators for prognosis and therapeutic outcomes of B-ALL patients. The impact of certain genetic aberrations on prognosis was also confirmed in our institute (**Figures 3A–C**, **Figures S4A–C**, and **Tables S2** and **S3**). However, for patients without any typical genetic changes, markers for prognosis prediction are limited. We identified CD123 expression as an independent marker in these patients. **Figures 3D–F** showed that CD123 expression was strongly correlated with the clinical outcomes of patients in absence of genomic abnormalities in the CCLG-2008 cohort, with superior OS (p = 0.0004), EFS (p = 0.0006), and RFS (p < 0.0001) in patients with high CD123 as compared to those with low or negative CD123 expression. A similar association was also observed in the CCCG-2015 cohort, with longer EFS time in CD123 high patients compared to those with low or negative CD123 expression (p = 0.0222). However, statistical significance was not achieved in OS (p = 0.4417) and RFS (p = 0.0707; **Figures S4D–F**).

The Univariate and multivariate survival analysis were also performed to evaluate the prognostic value of CD123 in patients without prognosis-defining genomic abnormalities. As shown in **Table 5**, high CD123 expression was an independent prognosis factor for favorable EFS (HR, 0.342; 95% CI, 0.135–0.868; p = 0.024) and RFS (HR, 0.226; 95% CI, 0.085–0.599; p = 0.003) in the CCLG-2008 cohort. Consistently, high CD123 expression was independently associated with superior EFS (HR, 0.508; 95% CI, 0.274–0.944; p = 0.032) and RFS (HR, 0.367; 95% CI, 0.139–0.971; p = 0.043) in the CCCG-2015 cohort (**Tables S3** and **S4**).

**Table 5:** Prognostic factors for therapeutic outcomes of patients lacking prognosis-defining genomic abnormalities in CCLG-ALL-2008 cohort.

Overall Survival									
		Univariate Cox’s regression				Multivariate Cox’s regression			
Variate	Category	OR	95% CI		p value	OR	95% CI		p value
			Lower	Upper			Lower	Upper	
CD123	Negative				0.001				**0.030**
	High	0.241	0.110	0.530	0.000	0.448	0.144	1.389	0.164
Age	<1				0.004				**0.018**
	1–10	0.045	0.006	0.355	0.003	0.026	0.002	0.341	**0.005**
	>10	0.099	0.011	0.855	0.036	0.039	0.002	0.669	**0.025**
WBC (×10^9^/L)	WBC<20				0.046				0.828
	50<WBC<100	2.915	1.156	7.350	0.023	1.459	0.349	6.099	0.605
	WBC>100	3.625	1.067	12.335	0.039	2.034	0.379	10.921	0.408
Risk group	LR				0.036				0.801
	IR	2.398	0.871	6.601	0.090	0.995	0.305	3.247	0.994
	HR	3.866	1.376	10.863	0.010	1.389	0.362	5.336	0.632
Day 33 MRD level	0.01–1%	2.123	0.918	4.905	0.078	2.039	0.776	5.358	0.148
**Event-free Survival**									
		**Univariate Cox’s regression**				**Multivariate Cox’s regression**			
**Variate**	**Category**	**OR**	**95% CI**		**p value**	**OR**	**95% CI**		**p value**
			**Lower**	**Upper**			**Lower**	**Upper**	
CD123	Negative				0.001				**0.044**
	High	0.331	0.183	0.599	0.000	0.342	0.135	0.868	**0.024**
Age	<1				0.066	0.880	0.282	2.747	0.826
	1–10	0.125	0.017	0.924	0.042			
Steroid resistance		2.247	1.187	4.253	0.013	1.051	0.331	3.336	0.933
Risk group	LR				0.000				0.590
	HR	4.024	1.921	8.429	0.000	1.556	0.422	5.744	0.507
Day 15 BM remission	M3	1.986	1.036	3.808	0.039	1.531	0.519	4.521	0.440
Day 33 MRD level	<0.01%				0.001				0.294
	0.01–1%	2.534	1.341	4.785	0.004	1.918	0.839	4.383	0.123
	>1%	5.955	1.969	18.008	0.002	1.401	0.228	8.602	0.716
Week 12 MRD level	>0.1%	3.511	1,597	7.722	0.002	2.446	0.861	6.948	0.093
**Relapse-free Survival**									
		**Univariate Cox’s regression**				**Multivariate Cox’s regression**			
**Variate**	**Category**	**OR**	**95% CI**		**p value**	**OR**	**95% CI**		**p value**
			**Lower**	**Upper**			**Lower**	**Upper**	
CD123	Negative				0.000				**0.007**
	Low	0.452	0.198	1.032	0.059	0.613	0.209	1.800	0.373
	High	0.219	0.108	0.443	0.000	0.226	0.085	0.599	**0.003**
Risk group	IR	2.215	0.931	5.269	0.072	0.856	0.297	2.467	0.773
Day 33 MRD level	<0.01%				0.003				**0.031**
	0.01–1%	4.141	1.843	9.305	0.001	3.206	1.296	7.933	**0.012**
Week 12 MRD level	>0.1%	3.013	1.226	7.404	0.016	2.694	0.858	8.461	**0.090**

Bold numbers represent that p value reaches statistical significance.

## Discussion

Several studies have previously described the pervasive CD123 expression in B-ALL (11, 22, 23). Our study confirmed that 75 and 67.7% pediatric B-ALL bone marrow blasts were CD123 positive (>20%) in the CCLG-ALL-2008 and CCCG-ALL-2015 cohorts, respectively. One recent study investigated the influence of CD123 expression on clinical outcomes of adult B-ALL, but no correlation was found between CD123 prevalence and leukemia-free survival or overall survival in adult B-ALL patients (23). However, B-ALL was predominantly a malignant disorder in childhood and to date the prognostic significance of CD123 in pediatric B-ALL remains unclear. In the current study, we assessed the association of CD123 expression with clinicopathological factors and treatment outcomes in pediatric B-ALL. Our data demonstrated that high CD123 expression was associated with favorable OS, EFS, and RFS of pediatric B-ALL, especially in patients without prognosis-defining genetic aberrations.

In order to improve the treatment outcomes of pediatric ALL, various regimens have been developed in China during the past 10 years. CCLG-ALL-2008 was a nationwide collaboration program involving 10 major teaching hospitals from different regions of China including our institute. A multicenter study showed that by applying the CCLG-ALL-2008 protocol, the estimate of 5‐year OS and EFS for pediatric B-ALL was 85.3 ± 0.8% and 79.9 ± 0.9% from 2008 to 2012 (21). In our single-center, the 5-year OS and EFS were 85.7 ± 1.9% and 73.8 ± 2.4%, which were similar to the multicenter data.

The CCCG-ALL-2015 protocol modified from St Jude Children’s Research Hospital Total XV for children with newly diagnosed ALL was launched in China since January 2015. Twenty institutions from different regions in China participated in this multicenter study. In the current study, a total of 648 newly diagnosed B-ALL patients from our institute were included. The estimated 5-year OS and EFS in the CCCG-ALL-2015 regimen was 94.1 ± 1.4% and 77.4 ± 2.7%, which showed a substantial improvement over CCLG-ALL-2008.

Compared with CCLG-2008 regimen, increased frequency and doses of asparaginase was applied in CCCG-ALL-2015 protocol. Intensified asparaginase usage was shown to improve the outcome in previous trials (24). In patients with hypersensitivity reactions to native *Escherichia coli* asparaginase, erwinia asparaginase was used at high and frequent doses as insufficient doses led to inferior outcomes (25, 26). Also, intensive asparaginase treatment was important to control CNS leukemia (26). Another modification of CCCG-ALL-2015 protocol was the its emphasis on the minimal residual disease (MRD) monitoring. In CCCG-ALL-2015 protocol, MRD monitoring was performed on Day 19 and Day 46 and risk stratification can be adjusted timely based on the results of sequential MRD measurement, which results in the precise risk assignment and efficient treatment.

Risk stratification and risk-based therapies have major influences on disease relapse and therapy-related toxicity. Although the criteria for risk stratification had minor changes between the two protocols, the frequency of CD123 expression was significantly correlated with risk stratification in both regimen protocols. As a factor commonly used in routine MRD measurement for ALL patients in our institute, the percentage of CD123 expression at initial diagnosis was easily accessible and could provide more information for risk assessment in pediatric B-ALL.

Our study also showed patients with a high percentage of CD123 expression had lower risks of mortality or relapse. Superior treatment outcomes were achieved in patients with high CD123 expression, displaying remarkably better OS, EFS, and RFS rates than those with low or negative CD123 expression. By univariate and multivariate analysis, a high percentage of CD123 expression on blasts was identified as an independent favorable prognostic factor in pediatric B-ALL. A retrospective analysis of 194 patients with B-ALL showed abnormal CD123 expression indicated a poorer OS and EFS (27). This discrepancy might be due to approximately 75% of patients in their center were adult B-ALL patients. The treatment outcome of adult B-ALL was much poorer as compared to pediatric B-ALL, with reported cure rates seldom exceeding 40% (28). These two age groups also differ in genetic landscape, regimens utilization, comorbidities, and treatment side effects (29, 30). One study compared the genetic landscape between pediatric and adult B-ALL and found adult patients had more alterations of B-cell development genes as compared to pediatric patients (31). It is likely that the CD123, as a critical component of the IL-3 signaling complex with known roles in B-cell survival (32), might have a differential expression pattern, even play different functional roles between pediatric and adult B-ALL patients.

Compelling evidence showed that aberrant CD123 expression was associated with blast proliferation, chemotherapy resistance, and relapse in AML patients. Patients with blasts overexpressing CD123 tended to have a poorer outcome (33). CD123-overexpressing AML blasts exhibited higher cell cycling activity and increased anti-apoptotic activity (13, 34). Approaches targeting CD123 have shown promising therapeutic results in different types of myeloid leukemia (35–37). Also, a handful of clinical trials were underway to investigate the efficacy CAR-123 in hematopoietic malignancies (38). Novel strategy such as Dual CART19/123 therapeutic strategy has shown impressive results in the mouse model (39).

Several studies suggested different IL-3/CD123 signaling between myeloid and lymphoid cells. A study in AML provided evidence that CD123 positive cells isolated from Fanconi anemia patients with AML exhibited increased IL-3 responsiveness (40). Compared with AML blasts with normal IL-3Rα expression, AML blasts with elevated IL-3Rα expression showed robust Stat5 activation, a key transcription factor in IL-3 signaling, after IL-3 stimulation (13), suggesting the expression level of IL-3Rα is essential in IL-3-mediated AML cell proliferation. By contrast, a previous study demonstrated that IL-3-responsive B lymphopoiesis was specifically found in cells with low or undetectable IL-3 Rα protein expression (41). Another study showed that in IL-3 responsive leukemic B-cell precursors (BCPs), low receptor (CD123) occupancy was sufficient for growth stimulation by rIL-3 (19). In comparison, ligand binding assays showed very few IL-3 binding sites per cell on IL-3 unresponsive leukemic BCPs. These two investigations suggest the high binding affinity of IL-3 to the IL-3 receptor (CD123 and CD131), other than the high expression level of CD123, plays a more important role in IL-3 responsiveness for B lymphoid (leukemic) cells (19, 41).

Genomic abnormalities are drivers of oncogenesis. Certain cytogenetic abnormalities are of great clinical significance for risk stratification by conferring different prognosis (42). In the present study, our data demonstrated that CD123 percentage was closely correlated with hyperdiploidy karyotype. Over 85% of cases with hyperdiploidy karyotype (Chromosome number 51–65) had a higher frequency of CD123 expression from our institute. This finding was consistent with the previous study in a cohort of 95 pediatric and 24 adult ALL patients (22). The gain of chromosome X, where CD123 is located, was a common event in pediatric ALL with a hyperdiploid karyotype (43), implying that the high CD123 expression in patients with hyperdiploidy might result from the increased gene dosage.

## Conclusions

Based on our large single-center pediatric B-ALL series, we concluded that high CD123 expression significantly correlates with favorable OS, EFS, and RFS of pediatric B-ALL, thus serving as a favorable prognostic factor for pediatric B-ALL patients. Notably, for B-ALL patients lacking prognosis-defining genetic aberrations, CD123 was identified as an independent favorable prognostic factor for EFS and RFS, thus providing a valuable marker for outcome prediction in these patients.

## Data Availability Statement

The raw data supporting the conclusions of this article will be made available by the authors, without undue reservation.

## Ethics Statement

The studies involving human participants were reviewed and approved by Children’s Hospital of Soochow University Institutional Ethics Committee. Written informed consent to participate in this study was provided by the participants' legal guardian.

## Author Contributions

SH and SX designed and directed the study. JP helped with statistical analysis and manuscript revising. ZL, XC, LG, and JinL were responsible for data collection and analysis. ZL drafted the manuscript. PX, JunL, and YW helped with the interpretation of results. HH, JQL, HY, and JieL helped collect and integrate all the clinical data. All authors contributed to the article and approved the submitted version.

## Funding

This work was supported by NSCF 81770193, 81700165, 81802499, and 81970163, Jiangsu projects (CXTDA2017014, BE2017659, and BE2017658), Suzhou projects (SYS2018075, SS201809, and SZZX201504), and National Clinical Research Center for Hematological Disorders.

## Conflict of Interest

The authors declare that the research was conducted in the absence of any commercial or financial relationships that could be construed as a potential conflict of interest.

## Supplementary Material

Supplementary Figure 1 CD123 expression in pediatric B-ALL patients from the CCLG-2008 and CCCL-2015 cohorts. **(A)** The plot of CD123 expression on leukemic blasts in pediatric B-ALL patients from the CCLG-2008 and CCCL-2015 cohorts. **(B)** The detailed statistical description of CD123 expression in both cohorts.

Supplementary Figure 2 Pediatric B-ALL with favorable clinical outcomes displayed a high CD123 expression in the CCCL-2015 cohort. Comparison of CD123 expression on leukemic blasts between survivors and non-survivors **(A)**, event-free and with-event patients (events including drug resistance, treatment abandonment, relapse, and HSCT) during treatment **(B)**, and relapsed or non-relapsed B-ALL **(C)** in the CCCG-ALL-2015 cohort; **(D)** Detailed amounts of patients and mean CD123 percentage in each group from A-C. **, p<0.01.

Supplementary Figure 3 High CD123 expression correlates with favorable clinical outcomes of pediatric B-ALL treated with CCCG-ALL-2015 protocol. Comparison of OS **(A)**, EFS **(B)**, and RFS **(C)** among CD123high, CD123low, and CD123neg groups in the CCCG-ALL-2015 cohort; (D) The detailed number of event occurrences and cases under observation in the CD123high, CD123low, and CD123neg groups in every follow-up year. Estimated 5-year survival rates in each group were calculated; Comparison of OS **(E)**, EFS **(F)**, and RFS **(G)** between patients with over or less than 50% blasts expressing CD123; Comparison of OS **(H)**, EFS **(I)**, and RFS **(J)** between patients positively- or negatively expressing CD123.

Supplementary Figure 4 High CD123 expression conferred favorable clinical outcomes in pediatric B-ALL without prognosis-defining genomic abnormalities in the CCCG-ALL-2015 cohort. Kaplan-Meier analysis of overall OS **(A)**, EFS **(B)**, and RFS **(C)** across patients with different genetic abnormalities in the CCCG-ALL-2015 cohort; Comparison of OS (D), EFS (E), and RFS (F) according to the CD123 expression among patients without prognosis-defining genetic aberrations in the CCCG-ALL-2015 cohort.

## Abbreviations

ALL, Acute lymphoblastic leukemia; AML, Acute myeloid leukemia; LSC, Leukemic stem cell; CCLG, Chinese Children’s Leukemia Group; CCCG, Chinese Children’s Cancer Group; OS, Overall survival; EFS, Event-free survival; RFS, Relapse-free survival; WBC, White blood cell; MRD, Minimal residual disease; HSCT, Hematopoietic Stem Cell Transplantation; CAR, Chimeric antigen receptor.
