# Supplementary figures and images for: High Expression of Interleukin-3 Receptor Alpha Chain (CD123) Predicts Favorable Outcome in Pediatric B-Cell Acute Lymphoblastic Leukemia Lacking Prognosis-Defining Genomic Aberrations
Source: Front Oncol. 2021 Mar 16;11:614420. doi: 10.3389/fonc.2021.614420 (PMC8008053; doi:10.3389/fonc.2021.614420)

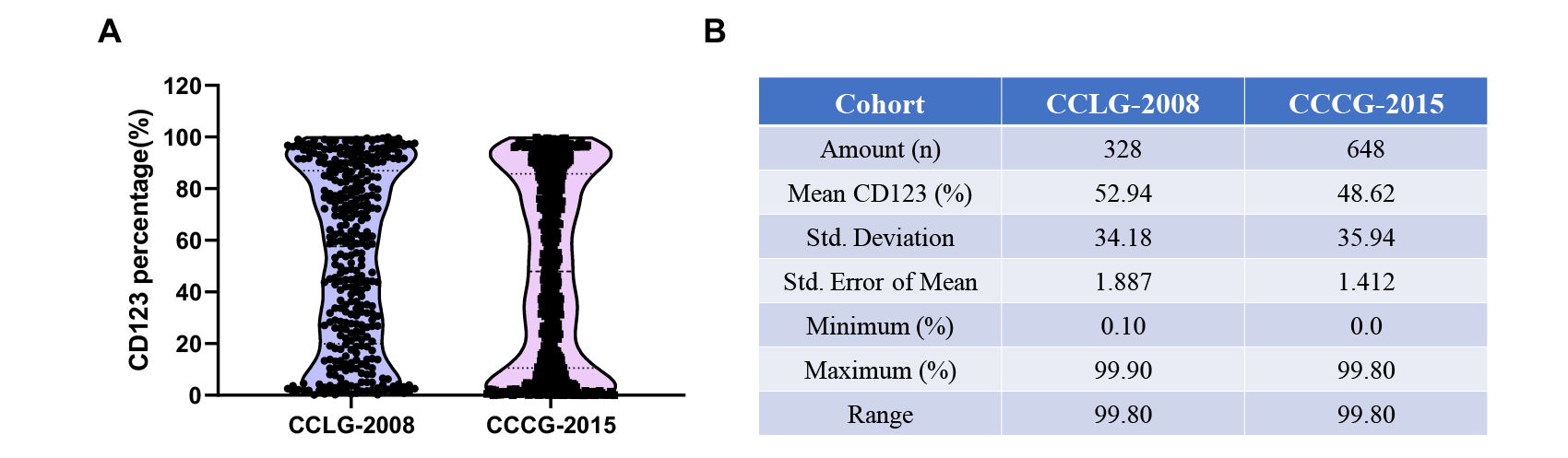

Supplement: Supplementary Figure 1 — CD123 expression in pediatric B-ALL patients from the CCLG-2008 and CCCL-2015 cohorts. (A) The plot of CD123 expression on leukemic blasts in pediatric B-ALL patients from the CCLG-2008 and CCCL-2015 cohorts. (B) The detailed statistical description of CD123 expression in both cohorts. [file Image_1.tif]

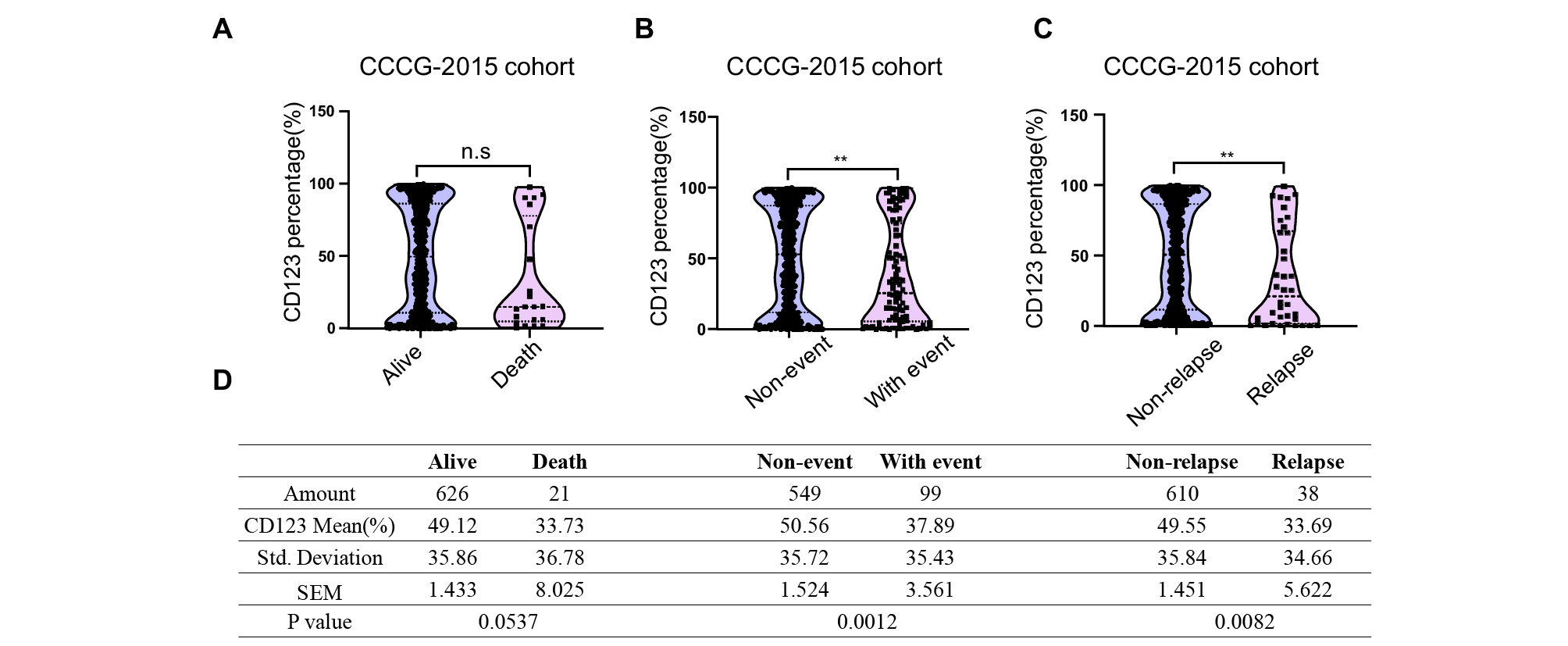

Supplement: Supplementary Figure 2 — Pediatric B-ALL with favorable clinical outcomes displayed a high CD123 expression in the CCCL-2015 cohort. Comparison of CD123 expression on leukemic blasts between survivors and non-survivors (A), event-free and with-event patients (events including drug resistance, treatment abandonment, relapse, and HSCT) during treatment (B), and relapsed or non-relapsed B-ALL (C) in the CCCG-ALL-2015 cohort; (D) Detailed amounts of patients and mean CD123 percentage in each group from A-C. **, p<0.01. [file Image_2.tif]

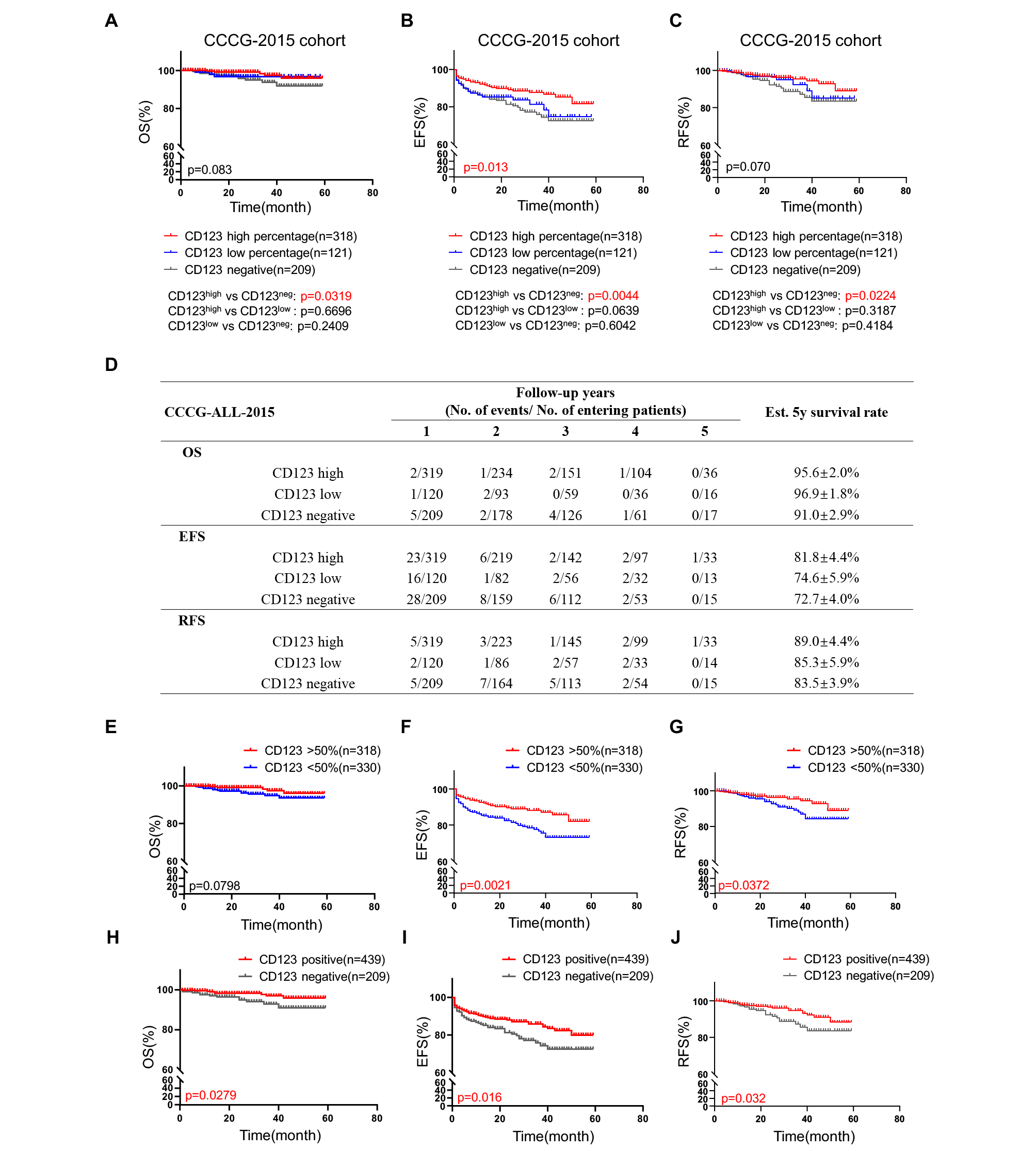

Supplement: Supplementary Figure 3 — High CD123 expression correlates with favorable clinical outcomes of pediatric B-ALL treated with CCCG-ALL-2015 protocol. Comparison of OS (A), EFS (B), and RFS (C) among CD123high, CD123low, and CD123neg groups in the CCCG-ALL-2015 cohort; (D) The detailed number of event occurrences and cases under observation in the CD123high, CD123low, and CD123neg groups in every follow-up year. Estimated 5-year survival rates in each group were calculated; Comparison of OS (E), EFS (F), and RFS (G) between patients with over or less than 50% blasts expressing CD123; Comparison of OS (H), EFS (I), and RFS (J) between patients positively- or negatively expressing CD123. [file Image_3.tif]

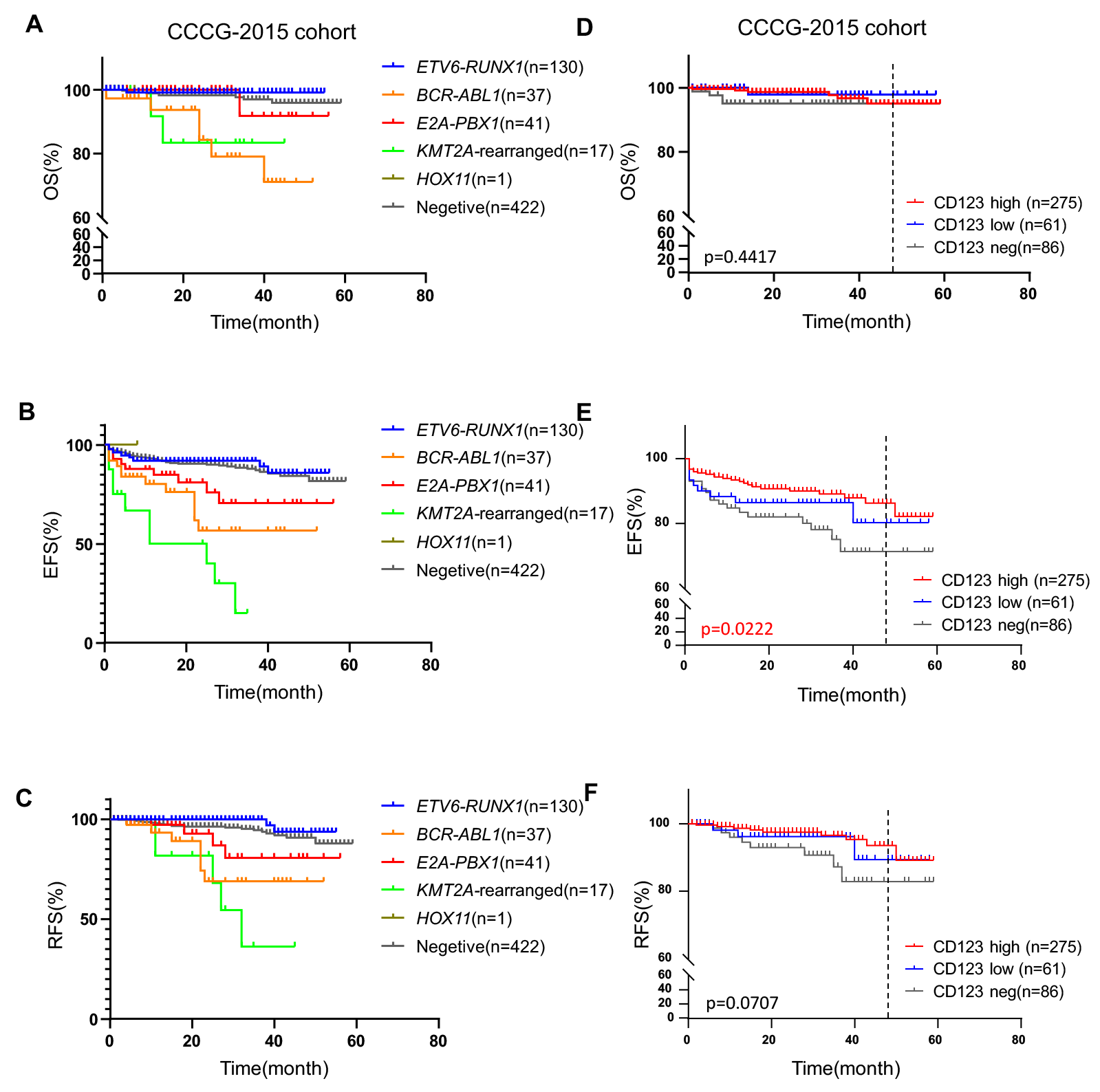

Supplement: Supplementary Figure 4 — High CD123 expression conferred favorable clinical outcomes in pediatric B-ALL without prognosis-defining genomic abnormalities in the CCCG-ALL-2015 cohort. Kaplan-Meier analysis of overall OS (A), EFS (B), and RFS (C) across patients with different genetic abnormalities in the CCCG-ALL-2015 cohort; Comparison of OS (D), EFS (E), and RFS (F) according to the CD123 expression among patients without prognosis-defining genetic aberrations in the CCCG-ALL-2015 cohort. [file Image_4.tif]
