# Supplementary material for: High Expression of Interleukin-3 Receptor Alpha Chain (CD123) Predicts Favorable Outcome in Pediatric B-Cell Acute Lymphoblastic Leukemia Lacking Prognosis-Defining Genomic Aberrations
Source: Front Oncol. 2021 Mar 16;11:614420. doi: 10.3389/fonc.2021.614420 (PMC8008053; doi:10.3389/fonc.2021.614420)
Supplement: Supplementary file 5 [file Table_1.docx]

**Supplement Table S1 The oligonucleotide sequence of multiplex RT-PCR for fusion detection.**

| **Reation No.** | **Split-Out**  **Reaction** | **Primers 1. PCR** | | **Primers 2. PCR** | |
| --- | --- | --- | --- | --- | --- |
|  |  | **Name** | **Sequence 5’ to 3’** | **name** | **Sequence 5’ to 3’** |
| R1 | AB | CBFB:267U22 | TTTGAAGGCTCCCATGATTCTG | CBFBMYH:344U21 | TGGGCTGTCTGGAGTTTGATG |
|  | A | MYH11:2198L22 | AGGTCCCCTTCCAGCTTCTTCT | MYH11:2041L19 | TGAGCGCCTGCATGTTGAC |
|  | B | MYH11:1438L24 | GAGCTGGATGTTGAGAGTGGAGAT | MYH11:1387L20 | TCCTCGTCCAGCTGGTCTTG |
|  | CDE | MLL:3730U20 | CCGCCTCAGCCACCTACTAC | MLL:3751U20 | GGACCGCCAAGAAAAGAAGT |
|  | CDE | MLL:3955U24 | AGCACTCTCTCCAATGGCAATAGT | MLL:3996U24 | AGCAGATGGAGTCCACAGGATCAG |
|  | C | AFX1:29L20 | GGGTGACTGGCAGCACAGAT | AFX1:5L24 | GGTTTCTTCTTGGGGGCTTTAACT |
|  | D | AF6:279L21 | CCGCTGACATGCACTTCATAG | AF6:242L22 | GAGGACAGCATTCGCATATCAG |
|  | E | ELL:351L23 | ACACCGTGATCTTGTCCTGTATG | ELL:306L22 | TTCCCCATGACTGGAGACATAC |
|  | ABCDE | E2A:1075U21 | TTCTCGTCCAGCCCTTCTACC | E2A:1173U19 | CTACGACGGGGGTCTCCAC |
|  | ABCDE | E2A:1883L22 | TTTTCCTCTTCTCGCCGTTTCA | E2A:1844L19 | AGGTTCCGCTCTCGCACTT |
| R2 | ABCDEF | MLL:3730U20 | CCGCCTCAGCCACCTACTAC | MLL:3751U20 | GGACCGCCAAGAAAAGAAGT |
|  | ABCDEF | MLL:3955U24 | AGCACTCTCTCCAATGGCAATAGT | MLL:3996U24 | AGCAGATGGAGTCCACAGGATCAG |
|  | A | AF1P:273L22 | GGATACCTTTGCCATCTGTGTC | AF1P:255L22 | TGTCGGCTAAATCCCAAATCT |
|  | B | AF17:1937L21 | CCTCCAGGTCTGGCTCTGTGT | AF17:1834L23 | GTAGAGCCAGCCAGAGAAAACAC |
|  | C | AF10:2363L23 | CTGTTCTATGCTGGCTGCTACTG | AF10:2327L21 | AACTGCTGTTGCCTGGTTGAT |
|  | D | AF10:1109L22 | TTGCCCTCTGACCCTCTAGTCT | AF10:1074L23 | TTCCACTAGAGGTGTGTGCAGAG |
|  | E | AF10:728L22 | TGGACATTATCGGCACCATTAC | AF10:685L21 | GGCAAACTGAGCGCATGTTAC |
|  | F | MLL:391L23 | TTTAGAGGGGAAAACACAGATGG | MLL:333L22 | TCTAGGTCTCCCACGAGGTTTT |
|  | ABCDEF | E2A:1075U21 | TTCTCGTCCAGCCCTTCTACC | E2A:1173U19 | CTACGACGGGGGTCTCCAC |
|  | ABCDEF | E2A:1883L22 | TTTTCCTCTTCTCGCCGTTTCA | E2A:1884L19 | AGGTTCCGCTCTCGCACTT |
| R3 | A | PBX1:459L18* | GCCACGCCTTCCGCTAAC | PBX1:436L21 | CATGTTGTCCAGCCGCATCAG |
|  | D | SIL:24U19 | CGACCCCAACGTCCCAGAG | SIL:83U20 | CCCGCTCCTACCCTGCAAAC |
|  | D | TAL1:203L21 | CGGTCATCCTGGGGCATATTT | TAL1:179L20 | AGACCGGCCCCTCTGAATAG |
|  | B | HLF:1140L20 | GCCCAGCTCCTTCCTCAAGT | HLF:998L20 | CGCCTTGCCCAGTACTTGTC |
|  | C | TEL:871U23 | CACTCCGTGGATTTCAAACAGTC | TEL:944U23 | CTCATCGGGAAGACCTGGCTTAC |
|  | C | AML1A:1891L23 | AGCCGAGTAGTTTTCATCATTGC | AML1A:1772L21 | AGCACGGAGCAGAGGAAGTTG |
|  | ABCD | E2A:1075U21 | TTCTCGTCCAGCCCTTCTACC | E2A:1173U19 | CTACGACGGGGGTCTCCAC |
|  | ABCD | E2A:1883L22 | TTTTCCTCTTCTCGCCGTTTCA | E2A:1844L19 | AGGTTCCGCTCTCGCACTT |
| R4 | AB | AML1A:1863U21 | GATGGCACTCTGGTCACTGTG | AML1A:1885U20 | TGGCTGGCAATGATGAAAAC |
|  | B | AMLMDSEVI:2375L24 | CGATCTTCCTTTTGGTCCATATTC | AMLMDSEVI:2345L21 | CCCCAGGCATATTTGACTCTC |
|  | D | HOX11:590U20 | GGGCGTCAACAACCTCACTG | HOX11:617U22 | CTTCCCCTGGATGGAGAGTAAC |
|  | D | HOX11:857L21 | GTCTGCCGTCTCCACTTTGTC | HOX11:810L19 | GCGCATCGGTCATTTTGAG |
|  | A | ETO:327L23 | TCTCCTATCTCGGGTGAAATGTC | ETO:116L22 | CGTTGTCGGTGTAAATGAACTG |
|  | C | TLS:649U19 | GGTGGCGGTTATGGCAATC | TLS:690U19 | CAGCGGTGGCTATGGACAG |
|  | C | ERG:908L22 | GTTCATGTTGGGTTTGCTCTTC | ERG:833L19 | GGTGCCTTCCCAGGTGATG |
|  | ABCD | E2A:1075U21 | TTCTCGTCCAGCCCTTCTACC | E2A:1173U19 | CTACGACGGGGGTCTCCAC |
|  | ABCD | E2A:1883L22 | TTTTCCTCTTCTCGCCGTTTCA | E2A:1844L19 | AGGTTCCGCTCTCGCACTT |
| R5 | ABCDE | MLL:3730U20 | CCGCCTCAGCCACCTACTAC | MLL:3751U20 | GGACCGCCAAGAAAAGAAGT |
|  | ABCDE | MLL:3955U24 | AGCACTCTCTCCAATGGCAATAGT | MLL:3996U24 | AGCAGATGGAGTCCACAGGATCAG |
|  | A | AF4:1636L29 | GAATTTGAGTGAGTTTTTGAAGATGTATC | AF4:1606L25 | GTTTTTGGTTTGGGTTACAGAACT |
|  | D | AF9:1869L24 | CCAGATGTTTCCAGGTAACTCTGT | AF9:1818L24 | GAGCAAAGATCAAAATCAAATGTT |
|  | C | AF9:1498L22 | TTCGGCTGCCTCCTCTATTTAC | AF9:1466L26 | CTCCATTTCAGAGTCATTGTCGTTAT |
|  | E | AF1Q:660L22 | GCTTGAGAGGGAAGACAATGAG | AF1Q:580L20 | TGCTGGCAATGGGAGCTCTC |
|  | B | ENL:81L22 | CACCATCCAGTCGTGAGTGAAC | ENL:30L19 | GCGATGCCCCAGCTCTAAC |
|  | ABCDE | E2A:1075U21 | TTCTCGTCCAGCCCTTCTACC | E2A:1173U19 | CTACGACGGGGGTCTCCAC |
|  | ABCDE | E2A:1883L22 | TTTTCCTCTTCTCGCCGTTTCA | E2A:1844L19 | AGGTTCCGCTCTCGCACTT |
| R6 | A | BCR:1698U19 | CGCTCTCCCTCGCAGAACT | BCR:1777U19 | ACTGCCCGGTTGTCGTGTC |
|  | B | BCR:3060U23 | GAGTCACTGCTGCTGCTTATGTC | BCR:3128U22 | CACGTTCCTGATCTCCTCTGAC |
|  | ABC | ABL:661L20 | TTTTGGTTTGGGCTTCACAC | ABL:642L23 | ACACCATTCCCCATTGTGATTAT |
|  | CD | TEL:309U21 | GCTGCTGACCAAAGAGGACTT | TEL:343U24 | CCTCATTCAGGTGATGTGCTCTAT |
|  | D | PDGFR:2282L22 | CATAAGGGCTTGCTTCTCACTG | PDGFR:2090L22 | CATGGGGTCCACGTAGATGTAC |
|  | ABCD | E2A:1075U21 | TTCTCGTCCAGCCCTTCTACC | E2A:1173U19 | CTACGACGGGGGTCTCCAC |
|  | ABCD | E2A:1883L22 | TTTTCCTCTTCTCGCCGTTTCA | E2A:1844L19 | AGGTTCCGCTCTCGCACTT |
| R7 | A | DEK:870U24 | TGCCAATGTTAAGAAAGCAGATAG | DEK:892U21 | AGCAGCACCACCAAGAAGAAT |
|  | AB | CAN:2872L21 | GGCAAGGATTTGGTGTGAGAT | CAN:2642L20 | GTCTCTCGCTCTGGCACAAG |
|  | B | SET:468U23 | CACCCGAAATCAAATGGAAATCTG | SET:552U24 | TGAGGAACCAGAGAGCTTCTTTAC |
|  | C | AMLMDSEVI:4331U23 | CCACTAAGCGAAAGGATGAGAAG | AMLMDSEVI:4509U21 | CGTCGAATCAAGACCTGCTTC |
|  | C | AMLMDSEVI:4866L21 | TGCCGTGTTAGGTTTGCAGAC | AMLMDSEVI:4746L25 | GAACATAGAGGGCACTGACTGTAAG |
|  | ABC | E2A:1075U21 | TTCTCGTCCAGCCCTTCTACC | E2A:1173U19 | CTACGACGGGGGTCTCCAC |
|  | ABC | E2A:1883L22 | TTTTCCTCTTCTCGCCGTTTCA | E2A:1884L19 | AGGTTCCGCTCTCGCACTT |
| R8 | A | PLZF:1092U21 | CCACAAGGCTGACGCTGTATT | PLZF:1252U21 | GTGGGCATGAAGTCAGAGAGC |
|  | B | PML3:1211U19 | CAAGAAAGCCAGCCCAGAG | PML3:1370U21 | GCCAGTGTACGCCTTCTCCAT |
|  | C | PML3:861U19 | GTGCGCCAGGTGGTAGCTC | PML3:930U20 | CAGCGCGACTACGAGGAGAT |
|  | ABCE | RARA:540L19 | AAGCCCTTGCAGCCCTCAC | RARA:508L22 | CCCATAGTGGTAGCCTGAGGAC |
|  | DEF | NPM:290U25 | ACGAAGGCAGTCCAATTAAAGTAAC | NPM:403U21 | GGTTCAGGGCCAGTGCATATT |
|  | D | NPMALK:627L21 | CACACTTCAGGCAGCGTCTTC | NPMALK:590L19 | CTTGGGTCGTTGGGCATTC |
|  | F | MLF1:235L27 | AGCTCTCCCTCTACCATCAGAGATACT | MLF1:192L28 | AAAGGGTTCAGAAAAACTTCTTATCAT |
|  | ABCDEF | E2A:1075U21 | TTCTCGTCCAGCCCTTCTACC | E2A:1173U19 | CTACGACGGGGGTCTCCAC |
|  | ABCDEF | E2A:1883L22 | TTTTCCTCTTCTCGCCGTTTCA | E2A:1844L19 | AGGTTCCGCTCTCGCACTT |
