# Supplementary material for: High Expression of Interleukin-3 Receptor Alpha Chain (CD123) Predicts Favorable Outcome in Pediatric B-Cell Acute Lymphoblastic Leukemia Lacking Prognosis-Defining Genomic Aberrations
Source: Front Oncol. 2021 Mar 16;11:614420. doi: 10.3389/fonc.2021.614420 (PMC8008053; doi:10.3389/fonc.2021.614420)
Supplement: Supplementary file 6 [file Table_2.docx]

**Table S2 Correlation of CD123 expression with clinicopathological features of pediatric B-ALL patients treated with CCCG-ALL-2015 protocol.**

| **Feature** | **Category** | **Total** | **CD123** | | | | | | **p value** |
| --- | --- | --- | --- | --- | --- | --- | --- | --- | --- |
|  |  |  | **negative** | | **Low percentage** | | **High percentage** | |  |
| All |  | 648 | 209 | 32.3% | 120 | 18.5% | 319 | 49.2% |  |
| Gender |  |  |  |  |  |  |  |  | 0.567 |
|  | M | 366 | 116 | 31.7% | 73 | 19.9% | 177 | 48.4% |  |
|  | F | 282 | 93 | 33.0% | 47 | 16.7% | 142 | 50.4% |  |
| Age (years) |  |  |  |  |  |  |  |  | 0.519 |
|  | <1 | 7 | 1 | 14.3% | 3 | 42.9% | 3 | 42.9% |  |
|  | 1-10 | 590 | 190 | 32.2% | 108 | 18.3% | 292 | 49.5% |  |
|  | >10 | 51 | 18 | 35.3% | 9 | 17.6% | 24 | 47.1% |  |
| Chromosome |  |  |  |  |  |  |  |  | **<0.0001** |
| hyperdiploidy* | N | 358 | 146 | 40.8% | 73 | 20.4% | 139 | 38.8% |  |
|  | Y | 110 | 6 | 5.5% | 10 | 9.1% | 94 | 85.5% |  |
| Molecular abnormality |  |  |  |  |  |  |  |  | **<0.0001** |
|  | *TEL-AML1* fusion | 130 | 63 | 48.5% | 41 | 31.5% | 26 | 20% |  |
|  | *BCR-ABL1* fusion | 37 | 13 | 35.1% | 11 | 29.7% | 13 | 35.1% |  |
|  | *E2A-PBX1* fusion | 41 | 39 | 95.1% | 1 | 2.4% | 1 | 2.4% |  |
|  | *MLL* rearrangement | 17 | 8 | 47.1% | 5 | 29.4% | 4 | 23.5% |  |
|  | *HOX11* | 1 | 0 | 0% | 1 | 100% | 0 | 0% |  |
| WBC (×10^9^/L) |  |  |  |  |  |  |  |  | 0.344 |
|  | WBC<20 | 462 | 139 | 30.1% | 82 | 17.7% | 241 | 52.2% |  |
|  | 20<WBC<50 | 86 | 31 | 36% | 16 | 18.6% | 39 | 45.3% |  |
|  | 50<WBC<100 | 66 | 25 | 37.9% | 14 | 21.2% | 27 | 40.9% |  |
|  | WBC>100 | 34 | 14 | 41.2% | 8 | 23.5% | 12 | 35.3% |  |
| Risk group |  |  |  |  |  |  |  |  | **0.004** |
|  | SR | 348 | 90 | 26.3% | 68 | 19.5% | 190 | 54.1% |  |
|  | IR | 286 | 115 | 41.2% | 49 | 17.3% | 122 | 41.5% |  |
|  | HR | 14 | 4 | 28.6% | 3 | 21.4% | 9 | 50.0% |  |
| Day19 MRD level † |  |  |  |  |  |  |  |  | 0.363 |
|  | Negative | 498 | 166 | 33.3% | 90 | 18.1% | 242 | 48.6% |  |
|  | Positive | 139 | 38 | 27.3% | 25 | 18.0% | 76 | 54.7% |  |
| Day46 MRD level‡ |  |  |  |  |  |  |  |  | 0.902 |
|  | Negative | 606 | 198 | 32.7% | 110 | 18.2% | 298 | 49.2% |  |
|  | Positive | 13 | 4 | 30.8% | 3 | 23.1% | 6 | 46.2% |  |
| Steroid response§ |  |  |  |  |  |  |  |  | 0.912 |
|  | N | 642 | 207 | 32.2% | 119 | 18.5% | 316 | 49.2% |  |
|  | Y | 5 | 2 | 40.0% | 1 | 20.0% | 2 | 40.0% |  |
| Relapse |  |  |  |  |  |  |  |  | **0.039** |
|  | N | 610 | 190 | 31.1% | 113 | 18.5% | 307 | 50.3% |  |
|  | Y | 38 | 19 | 50.0% | 7 | 18.5% | 12 | 31.6% |  |
| Death |  |  |  |  |  |  |  |  | **0.044** |
|  | N | 627 | 197 | 31.4% | 117 | 18.7% | 313 | 49.9% |  |
|  | Y | 21 | 12 | 57.1% | 3 | 14.3% | 6 | 28.6% |  |

* No metaphase cell available for karyotyping in 180 patients.

† No specific marker suitable for MRD monitoring in 11 patients.

‡ No specific marker suitable for MRD monitoring in 11 patients and no MRD data available in 18 patients.

§ Undetermined drug response in 1 patients.

WBC, white blood cell; SR, standard risk; IR, intermediate risk; HR, high risk; MRD, minimal residual disease.

Bold values indicate that the p value reaches statistical significance.
