# Supplementary material for: High Expression of Interleukin-3 Receptor Alpha Chain (CD123) Predicts Favorable Outcome in Pediatric B-Cell Acute Lymphoblastic Leukemia Lacking Prognosis-Defining Genomic Aberrations
Source: Front Oncol. 2021 Mar 16;11:614420. doi: 10.3389/fonc.2021.614420 (PMC8008053; doi:10.3389/fonc.2021.614420)
Supplement: Supplementary file 7 [file Table_3.docx]

**Table S3 Mean CD123 percentage and 5-year survival rates of pediatric B-ALL patients with specific genetic abnormalities.**

| **CCLG-ALL-2008** |  | |  | |  | |  | |  | |  | |  |
| --- | --- | --- | --- | --- | --- | --- | --- | --- | --- | --- | --- | --- | --- |
| Genetic abnormality | ***TEL-AML1*** | | ***BCR-ABL1*** | | ***E2A-PBX1*** | | ***MLL r*** | | ***HOX11*** | | **Mixed** | | **Negative** |
| Amount | 61 | | 14 | | 22 | | 9 | | 19 | | 1 | | 200 |
| CD123 Mean (%) | 37.17 | | 45.64 | | 7.427 | | 45.83 | | 74.32 | | 70.30 | | 61.20 |
| Std. Deviation | 28.87 | | 27.90 | | 8.281 | | 34.87 | | 27.21 | | 0.000 | | 32.58 |
| Std. Error of Mean | 3.696 | | 7.457 | | 1.766 | | 11.62 | | 6.242 | | 0.000 | | 2.303 |
| 5-year OS | 93.4±3.2% | | 64.3±12.8% | | 90.9±6.1% | | 66.7±15.7% | | 100% | | 100% | | 83.5±2.6% |
| 5-year EFS | 83.6±4.7% | | 35.7±12.8% | | 86.4±7.3% | | 55.6±16.6% | | 94.7±5.1% | | 100% | | 71.0±3.2% |
| 5-year RFS | 90.2±3.8% | | 71.4±12.1% | | 90.9±6.1% | | 77.8±13.9% | | 94.7±5.1% | | 100% | | 80.5±2.8% |
| **CCCG-ALL-2015** | |  | |  | |  | |  | |  | |  | |
| Genetic abnormality | | ***TEL-AML1*** | | ***BCR-ABL1*** | | ***E2A-PBX1*** | | ***MLL r*** | | ***HOX11*** | | **Negative** | |
| Amount | | 130 | | 37 | | 41 | | 17 | | 1 | | 422 | |
| CD123 Mean (%) | | 28.21 | | 36.34 | | 5.276 | | 35.11 | | 27.20 | | 60.74 | |
| Std. Deviation | | 24.49 | | 27.66 | | 9.957 | | 31.68 | | 0.000 | | 34.78 | |
| Std. Error of Mean | | 2.156 | | 4.547 | | 1.555 | | 7.685 | | 0.000 | | 1.693 | |
| 5-year OS | | 99.2±0.8% | | 71.1±10.8% | | 91.7±8.0% | | 83.3±10.8% | | 100% | | 95.4±1.6% | |
| 5-year EFS | | 86.2±4.6% | | 56.7±10.1% | | 70.4±9.0% | | 14.1±11.9% | | 100% | | 79.7±3.5% | |
| 5-year RFS | | 93.6±4.4% | | 68.9±10% | | 80.8±9.1% | | 36.4±18.9% | | 100% | | 87.9±3.6% | |
