# Supplementary material for: High Expression of Interleukin-3 Receptor Alpha Chain (CD123) Predicts Favorable Outcome in Pediatric B-Cell Acute Lymphoblastic Leukemia Lacking Prognosis-Defining Genomic Aberrations
Source: Front Oncol. 2021 Mar 16;11:614420. doi: 10.3389/fonc.2021.614420 (PMC8008053; doi:10.3389/fonc.2021.614420)
Supplement: Supplementary file 8 [file Table_4.docx]

**Table S4 Prognostic factors for therapeutic outcomes of pediatric B-ALL patients lacking prognosis-defining genetic abnormalities in CCCG-ALL-2015 cohort.**

| **Overall Survival** | | | | | | |  |  | | | | | | |
| --- | --- | --- | --- | --- | --- | --- | --- | --- | --- | --- | --- | --- | --- | --- |
|  |  | **Univariate Cox’s regression** | | | | |  | **Multivariate Cox’s regression** | | | | | | |
| Variate | Category | OR | 95% CI | | | p value |  | OR | | 95% CI | | | | p value |
|  |  |  | Lower | Upper | |  |  |  |  | Lower | | Upper | |  |
| Age | <1 |  |  |  | | 0.027 |  |  | |  | |  | | **0.029** |
|  | 1-10 | 0.055 | 0.007 | 0.462 | | 0.008 |  | 0.046 | | 0.005 | | 0.446 | | **0.008** |
| Risk group | LR |  |  |  | | 0.056 |  |  | |  | |  | | 0.518 |
|  | HR | 14.045 | 1.551 | 127.167 | | 0.019 |  | 0.001 | | 0.000 | | 5.722E+193 | | 0.975 |
| Day46 MRD level | Positive | 21.526 | 2.523 | 183.676 | | 0.005 |  | 55446.804.914 | | 0.000 | | 4.248E+201 | | 0.962 |
| **Event-free Survival** | |  | | | | |  |  | | | | | | |
|  |  | **Univariate Cox’s regression** | | | | |  | **Multivariate Cox’s regression** | | | | | | |
| Variate | Category | OR | 95% CI | | | p value |  | OR | | 95% CI | | | | p value |
|  |  |  | Lower | Upper | |  |  |  |  | Lower | | Upper | |  |
| CD123 | Negative |  |  |  | | 0.027 |  |  | |  | |  | | 0.100 |
|  | High | 0.447 | 0.248 | 0.804 | | 0.007 |  | 0.508 | | 0.274 | | 0.944 | | **0.032** |
| Steroid resistance |  | 52.823 | 11.262 | 247.768 | | 0.000 |  | 2.638 | | 0.445 | | 15.629 | | 0.285 |
| Risk group | LR |  |  |  | | 0.000 |  |  | |  | |  | | **0.000** |
|  | HR | 20.391 | 8.592 | 48.390 | | 0.000 |  | 21.749 | | 5.885 | | 80.382 | | **0.000** |
| Day19 MRD level | Positive | 2.297 | 1.334 | 3.955 | | 0.003 |  | 1.559 | | 0.684 | | 3.555 | | 0.291 |
| Day46 MRD level | Positive | 38.198 | 15.414 | 94.661 | | 0.000 |  | n.s.* | |  | |  | |  |
| **Relapse-free Survival** | |  | | | | |  | |  | | | | | |
|  |  | **Univariate Cox’s regression** | | | | |  | | **Multivariate Cox’s regression** | | | | | |
| Variate | Category | OR | 95% CI | | | p value |  | OR | | 95% CI | | | | p value |
|  |  |  | Lower | Upper | |  |  |  |  | Lower | | Upper | |  |
| CD123 | Negative |  |  | |  | 0.088 |  | |  |  |  | | 0.123 | |
|  | High | 0.344 | 0.132 | | 0.893 | 0.028 |  | | 0.367 | 0.139 | 0.971 | | **0.043** | |
| WBC | WBC>100 | 5.131 | 1.148 | | 22.931 | 0.032 |  | | 2.541 | 0.534 | 12.105 | | 0.241 | |
| Risk group | IR | 2.327 | 0.944 | | 5.737 | 0.066 |  | | 2.453 | 0.958 | 6.281 | | 0.061 | |

* Degree of freedom reduced because of constant or linearly dependent covariates

The bold values indicate that the p value reaches statistical significance.
